# Supplementary material for: Profiling Genome-Wide DNA Methylation in Children with Autism Spectrum Disorder and in Children with Fragile X Syndrome
Source: Genes (Basel). 2022 Oct 4;13(10):1795. doi: 10.3390/genes13101795 (PMC9602177; doi:10.3390/genes13101795)
Supplement: Supplementary file 1 [file genes-13-01795-s001.zip › genes-1879766-supplementary.pdf]

genes

Supplementary Materials

Profiling Genome-Wide DNA Methylation in Children with Autism Spectrum Disorder and in Children with Fragile X Syndrome

Mittal Jasoliya [1,†], Jianlei Gu [2,†], Reem R. AlOlaby [3], Blythe Durbin-Johnson [4], Frederic Chedin [5,6] and Flora Tassone [1,7,*]

[1] Biochemistry and Molecular Medicine, University of California, Davis, Sacramento, CA 95817, USA
[2] School of Public Health: Biostatistics, Yale University, New Haven, CT 06510, USA
[3] College of Health Sciences, California Northstate University, Sacramento, CA 95757, USA
[4] Department of Public Health Sciences, Division of Biostatistics, University of California, Davis, CA 95616, USA
[5] Davis, Genome Center, University of California, CA 95616, USA
[6] Davis, Molecular & Cellular Biology, University of California, Davis, CA 95616, USA
[7] MIND Institute, UC Davis, Sacramento, CA 95817, USA
* Correspondence: ftassone@ucdavis.edu; Tel.: +916-703-0463
† These authors contributed equally to this work.

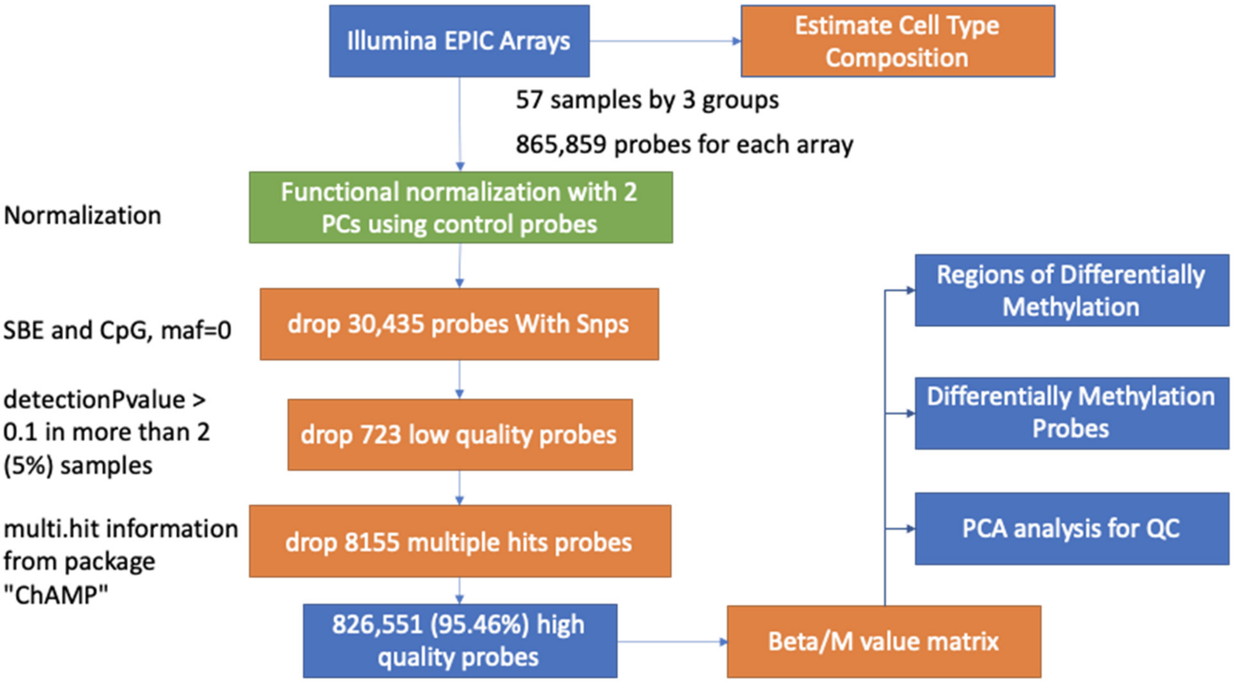

Figure S1. Analysis pipeline. Probes with a high detection p value (> 0.01), probes with a low success rate (missing in > 5% samples) and probes with SNPs were excluded for further analysis.

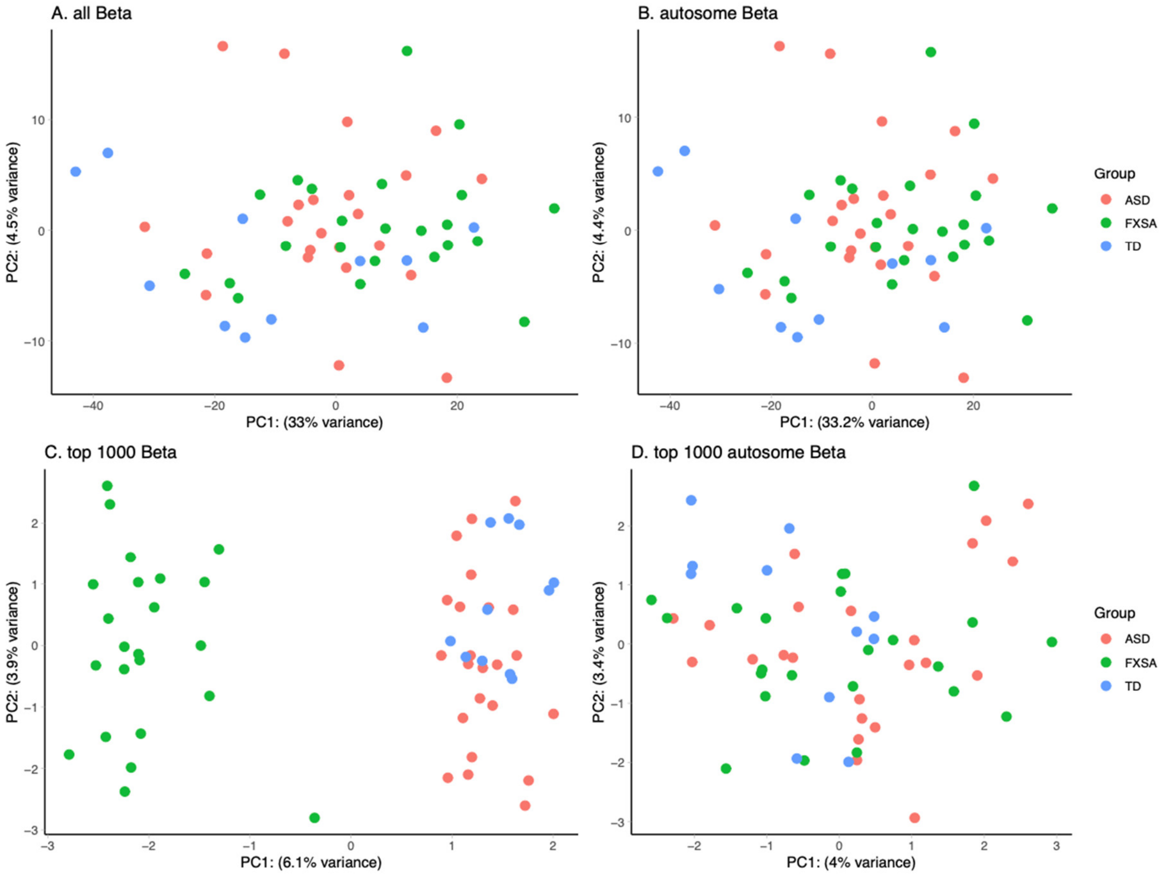

Figure S2. Principal component analysis plots (PC1 and PC2) for all normalized probes (Beta values). A) all CpG probes across samples. B) autosome CpG probes across samples. C) top 1000 most variable CpG probes across samples. D) top 1000 most variable autosome CpG probes across samples. PCA plots showing the samples from 3 disease status groups were jumbled all together.

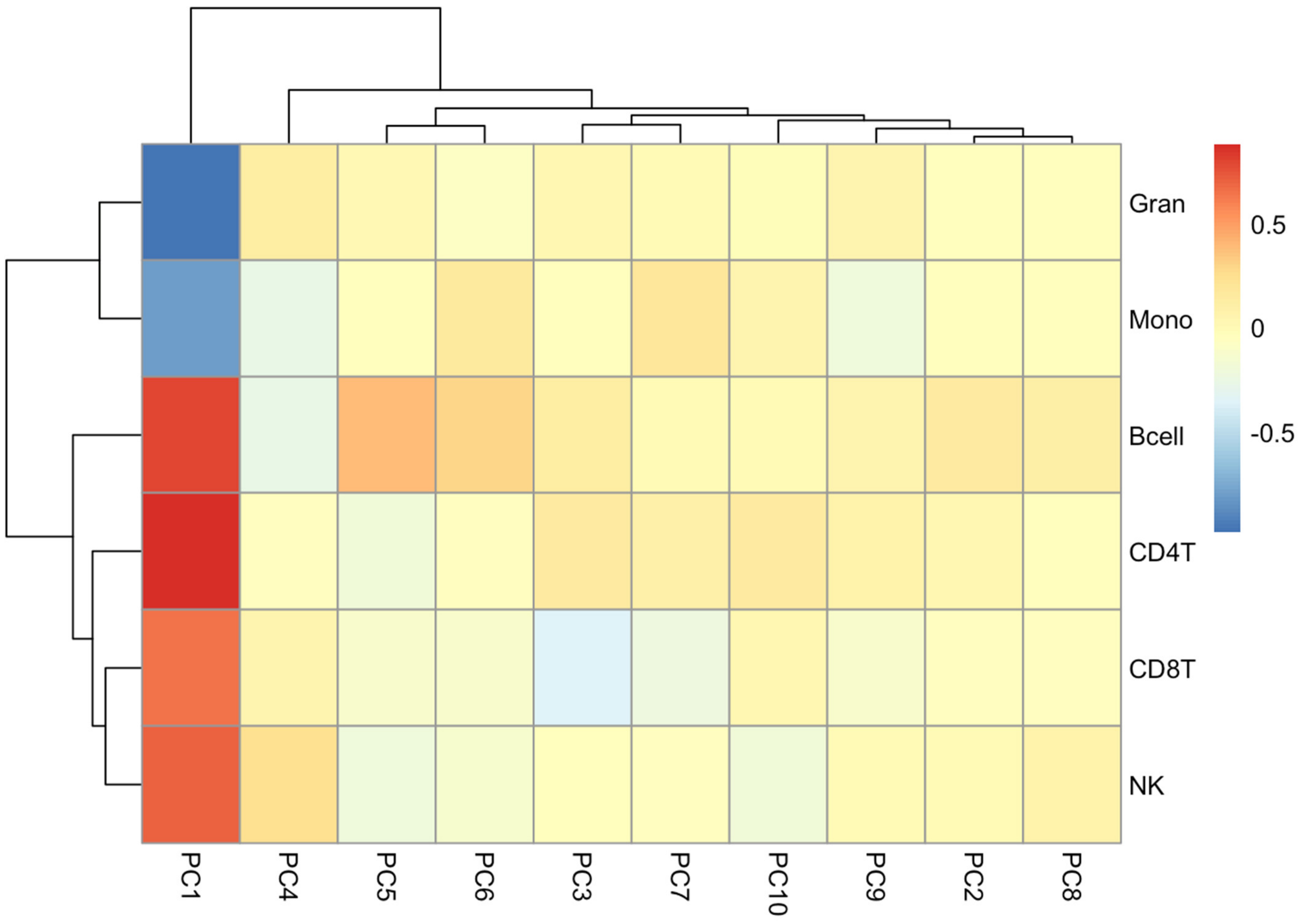

Figure S3. Correlation (Pearson) matrix between estimated cell type proportions and PCs. Positive correlations are represented in red, while negative correlations are in blue. The cell type proportions correlate strongly with PC1.

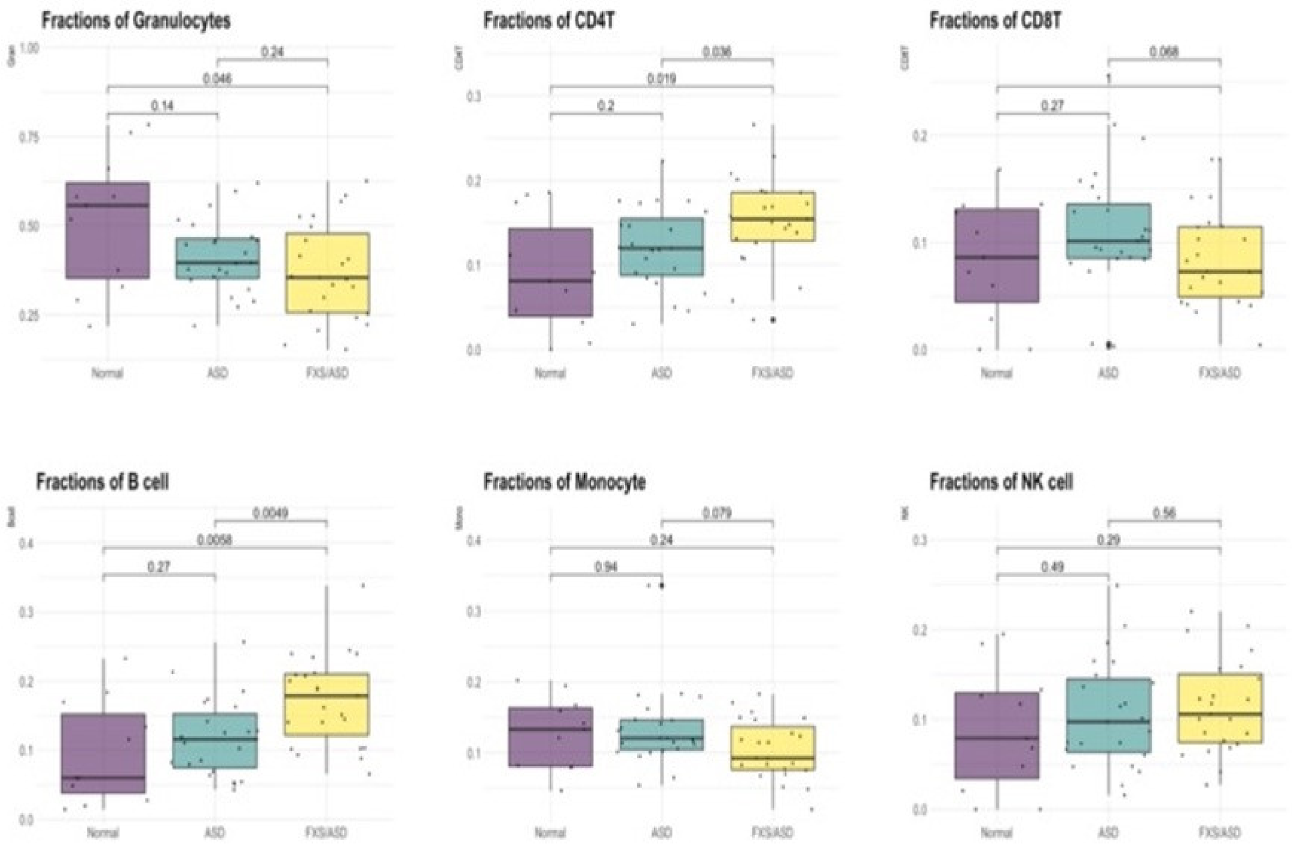

Figure S4. Cell type composition estimated by methylation data. Boxplots are used to visualize the cell type proportion by each disease group. We observed that the proportion of CD4T and CD8T were slightly higher than normal samples. Statistical significances of differences were tested by Wilcoxon test.

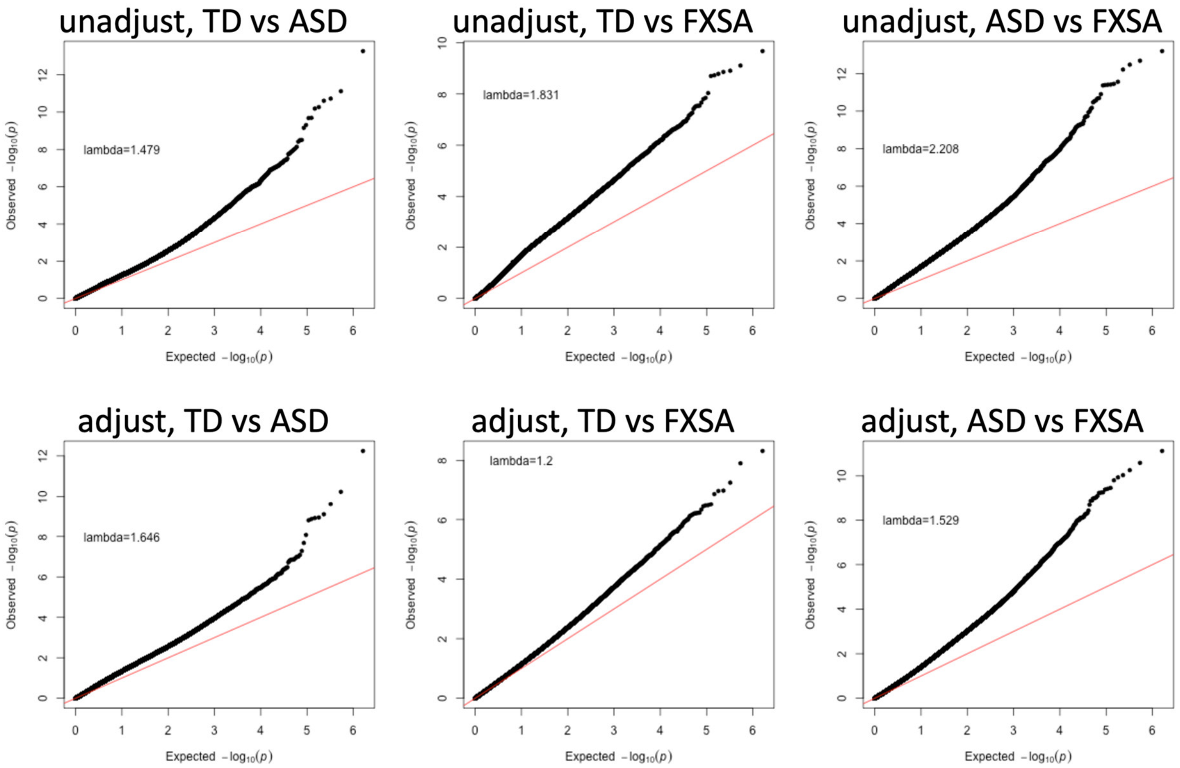

Figure S5. QQ-plots showing the association test inflation levels. Upper panel, before adjust cell type proportion, inflation coefficient ranges 1.479 – 2.208. Lower panel, after adjust cell type proportion, inflation coefficient ranges 1.200 – 1.646. The QQ-plots showing that after adjustment of cell type proportion, inflation is reduced.
